# Augmented reality for base plate component placement in reverse total shoulder arthroplasty: a feasibility study

**DOI:** 10.1007/s00402-020-03542-z

**Published:** 2020-07-26

**Authors:** Philipp Kriechling, Simon Roner, Florentin Liebmann, Fabio Casari, Philipp Fürnstahl, Karl Wieser

**Affiliations:** 1 Department of Orthopedics, Balgrist University Hospital, Zurich, Switzerland; 2 Computer Assisted Research and Development Group, Balgrist University Hospital, Zurich, Switzerland

**Keywords:** Shoulder, Reverse total shoulder, Augmented reality, Experimental

## Abstract

**Background:**

Accurate glenoid positioning in reverse total shoulder arthroplasty (RSA) is important to achieve satisfying functional outcome and prosthesis longevity. Optimal component placement can be challenging, especially in severe glenoid deformities. The use of patient-specific instruments (PSI) and 3D computer-assisted optical tracking navigation (NAV) are already established methods to improve surgical precision. Augmented reality technology (AR) promises similar results at low cost and ease of use. With AR, the planned component placement can be superimposed to the surgical situs and shown directly in the operating field using a head mounted display. We introduce a new navigation technique using AR via head mounted display for surgical navigation in this feasibility study, aiming to improve and enhance the surgical planning.

**Methods:**

3D surface models of ten human scapulae were printed from computed tomography (CT) data of cadaver scapulae. Guidewire positioning of the central back of the glenoid baseplate was planned with a dedicated computer software. A hologram of the planned guidewire with dynamic navigation was then projected onto the 3D-created models of the cadaver shoulders. The registration of the plan to the anatomy was realized by digitizing the glenoid surface and the base of the coracoid with optical tracking using a fiducial marker. After navigated placement of the central guidewires, another CT imaging was recorded, and the 3D model was superimposed with the preoperative planning to analyze the deviation from the planned and executed central guides trajectory and entry point.

**Results:**

The mean deviation of the ten placed guidewires from the planned trajectory was 2.7° ± 1.3° (95% CI 1.9°; 3.6°). The mean deviation to the planned entry point of the ten placed guidewires measured 2.3 mm ± 1.1 mm (95% CI 1.5 mm; 3.1 mm).

**Conclusion:**

AR may be a promising new technology for highly precise surgical execution of 3D preoperative planning in RSA.

## Introduction

Reverse total shoulder arthroplasty (RSA) was introduced by Grammont in 1985, [1] initially to treat rotator cuff tear arthropathy. Since then, the number of implanted RSA increased dramatically worldwide due to the expansion of indications [2]. RSA is by now increasingly indicated for patients suffering from glenohumeral osteoarthritis, rotator cuff tear, rheumatoid arthritis, fractures or as a viable revision option [3]. Despite promising results in terms of range of motion, pain relief and patient satisfaction, still, a considerable high number of complications remains. Scapular notching, instability and glenoid loosening are among the most common [2, 4, 5] and associated with mal-positioning of the glenoid component. The glenoid component should be placed inferior at the glenoid surface in neutral version and with neutral or slightly inferior tilt [6–8].

After soft tissue preparation of the glenoid, implant placement typically begins by placing a central guidewire with a surgical aiming instrument on the glenoid surface, followed by consecutive reaming for implant positioning. Achieving the correct position and orientation of the central guidewire is crucial for the correct positioning of the implant. The main challenge is the limited intraoperative view of the scapula and additionally, the difficult anatomical situation in case of glenoid deformations. Accurate implant positioning depends on correct two- or three-dimensional planning with a dedicated planning software. However, the surgical execution still depends mainly on the surgeon’s experience in the conventional setting [9]. Patient-specific instrumentation (PSI) and 3D computer-assisted optical tracking navigation (NAV) are valid options to improve the precision of implant positioning, but the technologies are associated with high costs, long production times and an increased effort of the surgical workflow [10].

Recently, the interest in mixed reality concepts for their use in surgery has increased dramatically [11]. Navigation accuracy is comparable with the systems mentioned above [12]. However, the application of the AR technology for the surgical navigation of glenoid component placement has not yet been described nor evaluated in the scientific literature. We aimed to improve and enhance the surgical planning and execution technology using AR and head-mounted display (HMD) in form of a first feasibility study.

We hypothesize that the application of AR is feasible for navigation of the central guidewire for glenoid component placement, allowing a highly precise surgical execution of the 3D preoperative planning.

## Materials and methods

### Trial design

The study was approved by the cantonal ethics committee under the number 2017-00874. We conducted an experimental trial employing ten 3D-printed scapulae. Each was individually segmented on existing human anatomy from human cadaver shoulders consisting of a full scapula and a proximal humerus. We ordered the scapulae under the number SHOU04 BM 061418 from Science Care of Florida (Coral Springs, Florida, United States of America).

### Scapulae models

Ten human cadavers were scanned with a computed tomography device (Siemens Somotom Edge Plus, Germany) in 0.5 mm slice increments. The scapulae were segmented with global thresholding and region growing using standard segmentation software (MIMICS version 23, Leuven, Belgium). The 3D models then were printed in our institution’s 3D printer EOS Formiga P100 (EOS GmBH, Munich, Germany) using PA2200 material.

**Fig. 1:**
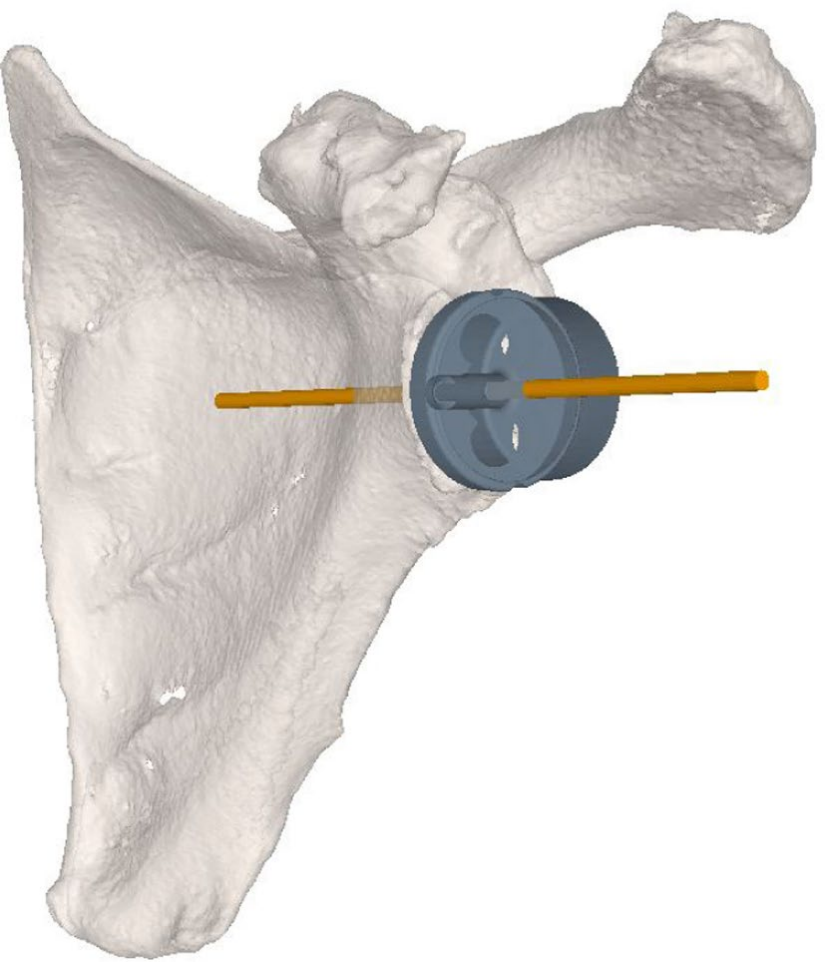
Preoperative: planned vector for guidewire positioning

Guidewire position and trajectory for the positioning of a glenoid baseplate 15 mm (BF Glenoid Trabecular Metal System; Zimmer Biomet ™, Warsaw, Indiana, USA) were planned on the 3D models by an experienced shoulder surgeon (K.W.) using a dedicated planning software (CASPA, Balgrist CARD, Zurich, Switzerland) (Fig. 1). The data were then adapted and converted using Unity Software (Unity Technologies, San Francisco, CA, USA, Version, 2019.1.7) and Microsoft Visual Studio (version Community 2017, Microsoft Corporation, Redmond, WA, USA) and then set up on the HoloLens.

### Surgical navigation

**Fig. 2:**
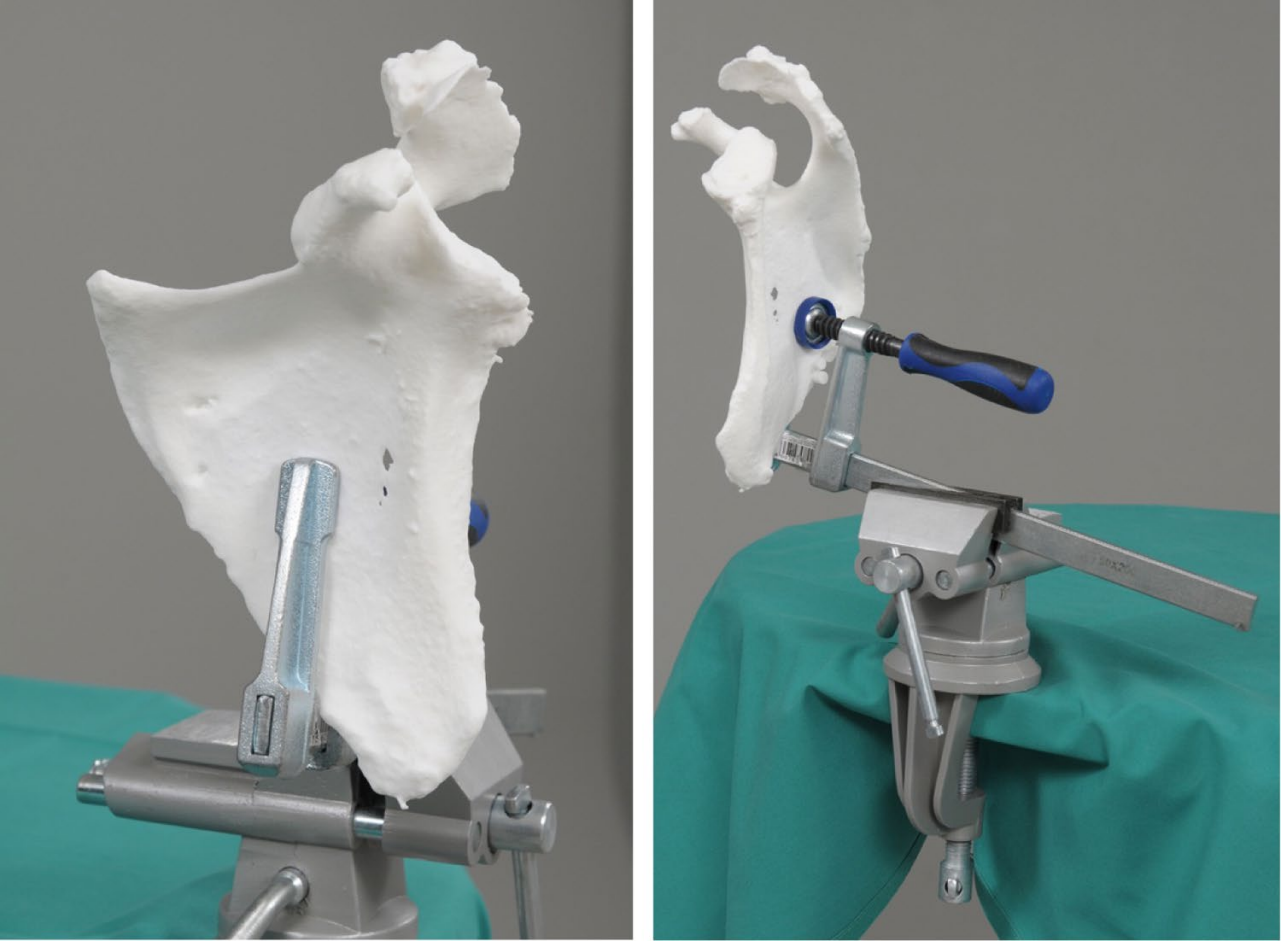
Placing the 3D model of the scapula on the work bench

**Fig. 3:**
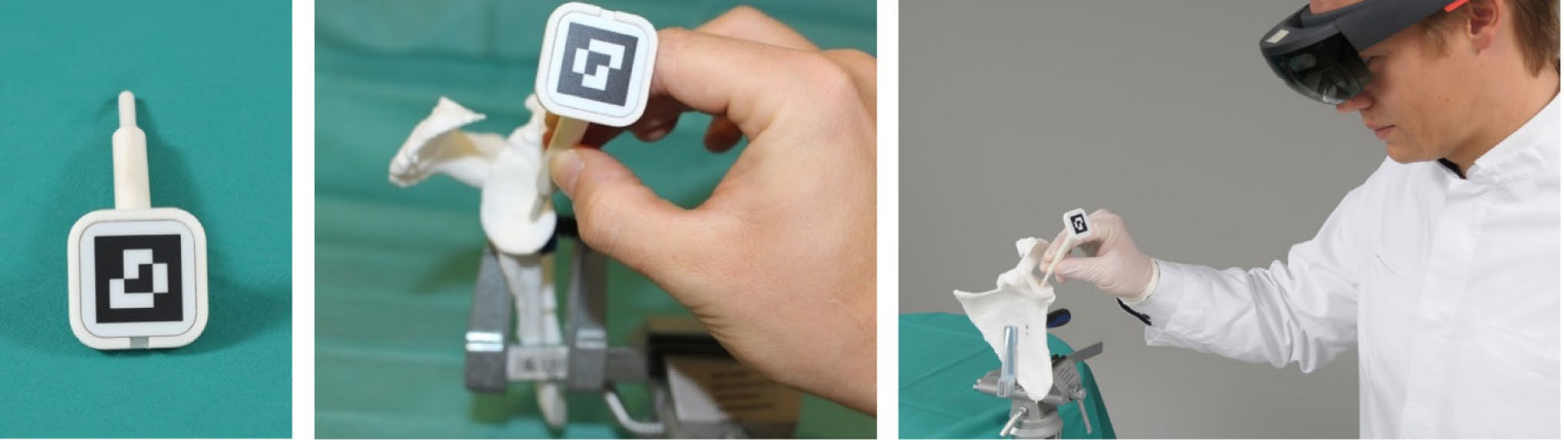
Optical tracking using a fiducial marker

**Fig. 4:**
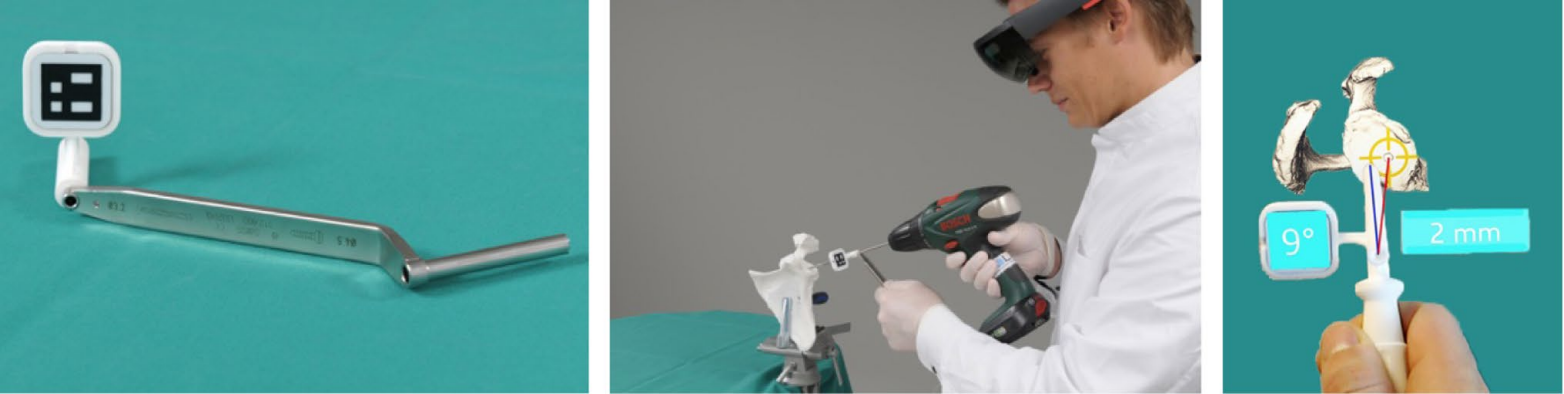
AR navigated drilling of the guidewire. The HoloLens shows the planned and the current trajectory

The head-mounted display (HMD) Microsoft HoloLens 1 (Microsoft, Redmond, WA, USA) was employed for the holographic navigation. The 3D printed scapula model was placed on a working bench using a clamp (Fig. 2). To register the position of the scapula in space, the surface of the scapulae was digitized with a custom-made fiducial marker and stereo tracking as described by Liebmann et al. [12] First, the rough orientation of the scapula was defined by marking the coracoid, acromion and glenoid. In real human surgery, this step could be done in the same manner or even through the intact skin. Subsequently, the glenoid surface and the coracoid base were continuously traced with the marker for fine adjustment (Fig. 3). For this step, we only allowed the glenoid surface and the coracoid base to be marked to simulate the limited accessibility of real surgery. The registration between preoperative planning and real anatomy was calculated from these data and the hologram of the planning was superimposed. In case of an insufficient surface tracing, the process could be repeated. To enable surgical navigation of the guidewire, a custom-made fiducial target instrument (Fig. 4) was attached to the drill sleeve, permitting constant position tracking by the HMD. Using the HMD, the surgeon was able to see the planned entry point, the planned target trajectory, the trajectory currently reached by the target instrument and the corresponding deviation in degrees and millimeters (Fig. 4). After the planned direction was achieved, the guidewire was drilled into the scapula using a drilling machine (PSR14,4 LI-2, Bosch AG, Gerlingen, Germany).

### Outcome parameters

**Fig. 5:**
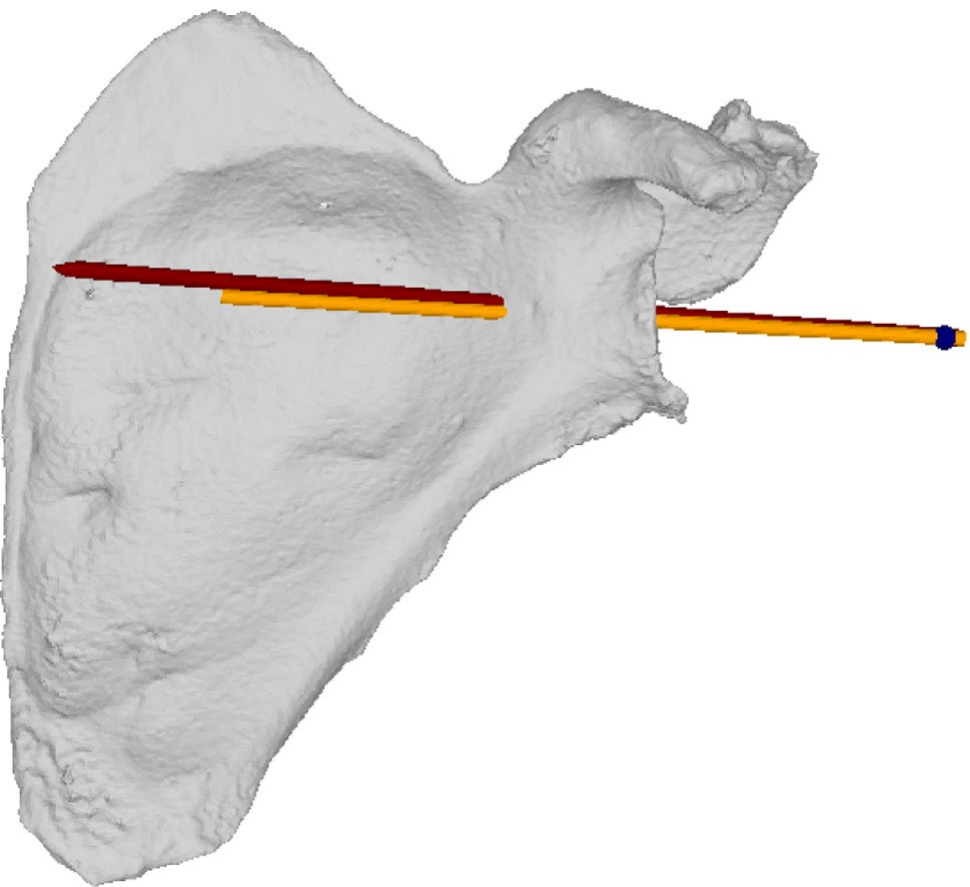
Postoperative: planned (orange) and achieved trajectory (red) for ID 3

After the intervention, the 3D printed scapulae were re-scanned by CT and segmented in the same manner as described above. The planned 3D model and the post-surgery 3D model were then aligned to each other in an automated fashion by applying the iterative closest point (ICP) surface registration method [13] using inhouse planning software (CASPA). We then calculated the deviation of the finally executed guidewire to the planned vector (3D angle) and the planned entry point as 3D distance (Fig. 5).

The 3D angular error (AE) was calculated using the direction vector of the planned ($$\overset{\lower0.5em\hbox{$\smash{\scriptscriptstyle\rightharpoonup}$}}{\text{A}}$$) and executed ($$\overset{\lower0.5em\hbox{$\smash{\scriptscriptstyle\rightharpoonup}$}}{\text{B}}$$) trajectories by applying the following formula:

$${\text{AE}} = \cos^{ - 1} \left( {\frac{{\overset{\lower0.5em\hbox{$\smash{\scriptscriptstyle\rightharpoonup}$}}{{\text{A} }} \cdot \overset{\lower0.5em\hbox{$\smash{\scriptscriptstyle\rightharpoonup}$}}{\text{B}}}}{{\left\| {\overset{\lower0.5em\hbox{$\smash{\scriptscriptstyle\rightharpoonup}$}}{\text{A}}} \right\|\left\| {\overset{\lower0.5em\hbox{$\smash{\scriptscriptstyle\rightharpoonup}$}}{\text{B}}} \right\| }}} \right).$$

The entry point error (TE) was calculated as Euclidean distance between the centers of the planned (*x*_1_, *y*_1_, *z*_1_) and achieved (*x*_2_, *y*_2_, *z*_2_) entry points using the following formula:

$${\text{TE}} = \sqrt {\left( {x_{1} - x_{2} } \right)^{2} + \left( {y_{1} - y_{2} } \right)^{2} + \left( {z_{1} - z_{2} } \right)^{2} } .$$

### Statistical methods

Calculations were done using SPSS v23.0 (IBM, New York, United States of America). Descriptive statistics are given as mean ± standard deviation (95% confidence interval).

## Results

All ten guidewires could be placed without any technical problems. The registration process by surface tracing was the most challenging part of the experiments and had to be repeated in mean 2–3 times per scapula to achieve correct alignment. The results of the accuracy evaluation are shown in Table 1.

**Table 1:** Displays the 3D vector, entry point of the ten scapulae and the values of mean, standard deviation (STD) and 95% confidence interval (CI)

ID	3D vector	Entry point
1	4.2	2.3
2	2.0	1.7
3	0.6	1.5
4	2.2	3.0
5	3.1	1.4
6	2.0	1.5
7	3.4	1.5
8	1.9	4.6
9	3.2	3.7
10	4.9	1.6
Mean ± STD (95% CI)	2.7° ± 1.3° (1.9°; 3.6°)	2.3 mm ± 1.1 mm (1.5 mm; 3.1 mm)

The mean 3D deviation angle of the ten placed wires measured 2.7° ± 1.3° (95% CI 1.9°; 3.6°) (Table 1).

The mean deviation to the entry point of the ten placed target wires measured 2.3 mm ± 1.1 mm (95% CI 1.5 mm; 3.1 mm) (Table 1).

## Discussion

Glenoid mal-positioning can worsen the outcome of RTSA. Deviation from the planned vector depends mainly on the surgeon’s experience and is secondarily complicated by the limited visibility of the scapula during surgery and glenoid deformation. To reduce the deviation error, new technologies such as PSI and 3D optical tracking navigation have been introduced. A completely new approach is the growing technology of AR. We, therefore, investigated the feasibility and accuracy of this new method for glenoid baseplate positioning. We were able to show that navigation of the guidewire positioning for the later placement of glenoid components using AR is feasible and accurate. We achieved an average planning deviation in 3D of 2.7° ± 1.3° (95% CI 1.9°; 3.6°) for the trajectory and 2.3 mm ± 1.1 mm (95% CI 1.5 mm; 3.1 mm) for the glenoid surface entry point. To the best of our knowledge, this is the first study demonstrating the approach of AR-navigated guidewire positioning in reverse total shoulder arthroplasty. Other authors described the ability to superimpose the scapula model over the situs in theatre. Berhouet et al. showed the overlay of a scapula over a model, but without navigation [14]. In 2018, Gregory et al. used AR while performing total shoulder arthroplasty by superimposing the anatomy of the scapula in situ by manually overlaying the model over the real anatomy [15].

The novel method yields acceptable applicability and high accuracy in the placement of the guidewire. Achieving the planned component positioning is particularly difficult in strongly deformed or even dysplastic glenoids. The optimal implant position is essential for a satisfying clinical outcome and longevity of the components [16]. To achieve high accuracy, PSI and optical tracking systems are currently available on the market as navigation systems [16]. A recently published meta-analysis of 227 shoulders showed a 2D accuracy of 2.7° ± 0.5° for the version and 1.9° ± 0.4° for the inclination with a deviation of the entry point of 1.1 mm ± 0.2 mm when using PSI. This was clearly superior to the standard freehand method (version 5.88° ± 1.10°, inclination 5.78° ± 0.98, entry point 2.04 mm ± 0.40) [10]. Currently, there is only one clinically randomized controlled trial that also shows superiority of PSI to freehand placement with deviation from planning for version of 4.3° vs. 6.9° and for inclination of 2.9° vs. 11.6°, both in favor of PSI [17].

A meta-analysis from 2015 with 247 shoulders from 5 studies showed an average 6.4° improvement of the version by NAV compared with standard component placement. The navigation advantage was 6.3° for TSA and 9.9° for RSA [18]. A current clinical application of Nashikkar in 60 subjects also shows the superiority of NAV. The deviation from the planning in the navigation group was 0.2° ± 4.8° and 1.4° ± 2.8° for version and inclination, respectively [19].

Our results have to be interpreted in consideration of the hardware limitations. Microsoft HoloLens is essentially produced as a multimedia entertaining device, hence lacking the necessary precision of high-end medical devices. We must highly value the applicability of the AR technology to solve medical problems and improve necessary procedures. The accuracy in placing glenoid components can be expected to even improve with newer AR devices, especially those dedicated to medical application.

Our study has, however, clear limitations. First, we used only 3D printed scapula models without soft tissue and a fully visible scapula. As a method-oriented approach, this does only represent an approximation to the intraoperative situation. Second, no control group was used. This is intended because the conventional freehand technique is highly dependent on the surgeon’s experience. To reduce the bias of the surgeon performing the procedure, a higher number of investigators would be necessary for both the freehand technique and the augmented reality navigation. Aware of this, we only used one investigator without control group to maintain the purpose of our work as a feasibility study. This leads to a high investigator dependency. Increasing the number of investigators performing the procedure should be subject to upcoming projects to reduce investigator dependency.

Using AR for guidewire placement, we could achieve similar good values as with NAV or PSI. There are, however, disadvantages of the different technologies. For PSI, an appropriate preparation time is required, because the guides must be produced in advance. Depending on the manufacturer, this can take several weeks and is currently still associated with high costs. With intraoperative optical tracking navigation, there are no production costs and a significantly shorter preoperative preparation time for surgical planning. However, the operation time for NAV is significantly longer compared to PSI due to device setup and the required intraoperative imaging [16, 20].

AR can combine the advantages of both technologies at a low cost. After preoperative planning and transfer of the data to the HMD, only the registration with an optical tracking marker is necessary intraoperatively. By tracking the surface with a marker, no intraoperative imaging is required, hence reducing radiation exposure. To improve AR usability for the operating theatre, further developments and studies are necessary. The next step would be to add the applicability of screw placement and transfer the application to human cadavers. Since the Microsoft HoloLens was designed as an entertainment device without primarily medical application purposes, an improvement of the AR navigation by future versions of the lens can be expected.

## Conclusion

AR offers a promising new technology for highly precise surgical execution of 3D preoperative planning in RSA.

## Abbreviations

AR: Augmented reality
CT: Computed tomography
HMD: Head-mounted display
NAV: 3D computer-assisted optical tracking navigation
PSI: Patient-specific instrumentation
RSA: Reverse total shoulder arthroplasty
